# Supplementary figures and images for: Dual function of perivascular fibroblasts in vascular stabilization in zebrafish
Source: PLoS Genet. 2020 Oct 26;16(10):e1008800. doi: 10.1371/journal.pgen.1008800 (PMC7644104; doi:10.1371/journal.pgen.1008800)

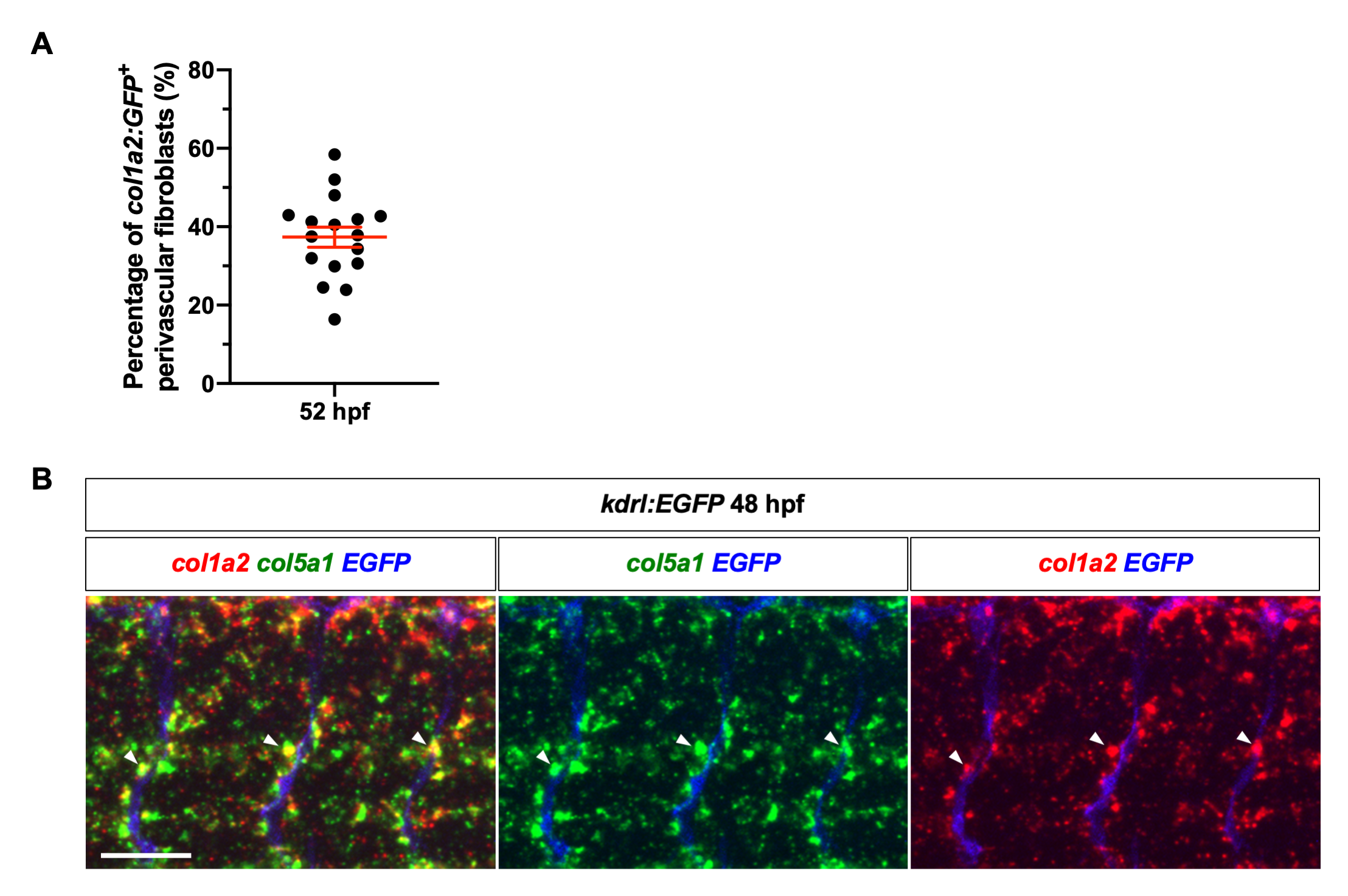

Supplement: S1 Fig — (A) Quantification of col1a2:GFP expression in perivascular fibroblasts in nkx3.1NTR-mCherry; col1a2:GFP embryos at 52 hpf from Fig 1B. Total number of ISV associated mCherry+ and GFP+ cells were counted, and GFP+mCherry+ perivascular fibroblasts were graphed as a proportion of all mCherry+ perivascular fibroblasts. Data are plotted as mean ± SEM. n = 17 embryos. (B) Co-expression of col1a2 and col5a1 in perivascular fibroblasts. kdrl:EGFP embryos at 48 hpf were co-labeled with col1a2 (red) and col5a1 (green) by double fluorescent in situ hybridization followed by immunofluorescence labeling using the GFP antibody (blue). Co-expression of col1a2 and col5a1 is observed in perivascular fibroblasts (arrowheads) along EGFP+ ISVs. n = 23 embryos. Scale bar: 50 μm. (TIF) [file pgen.1008800.s001.tif]

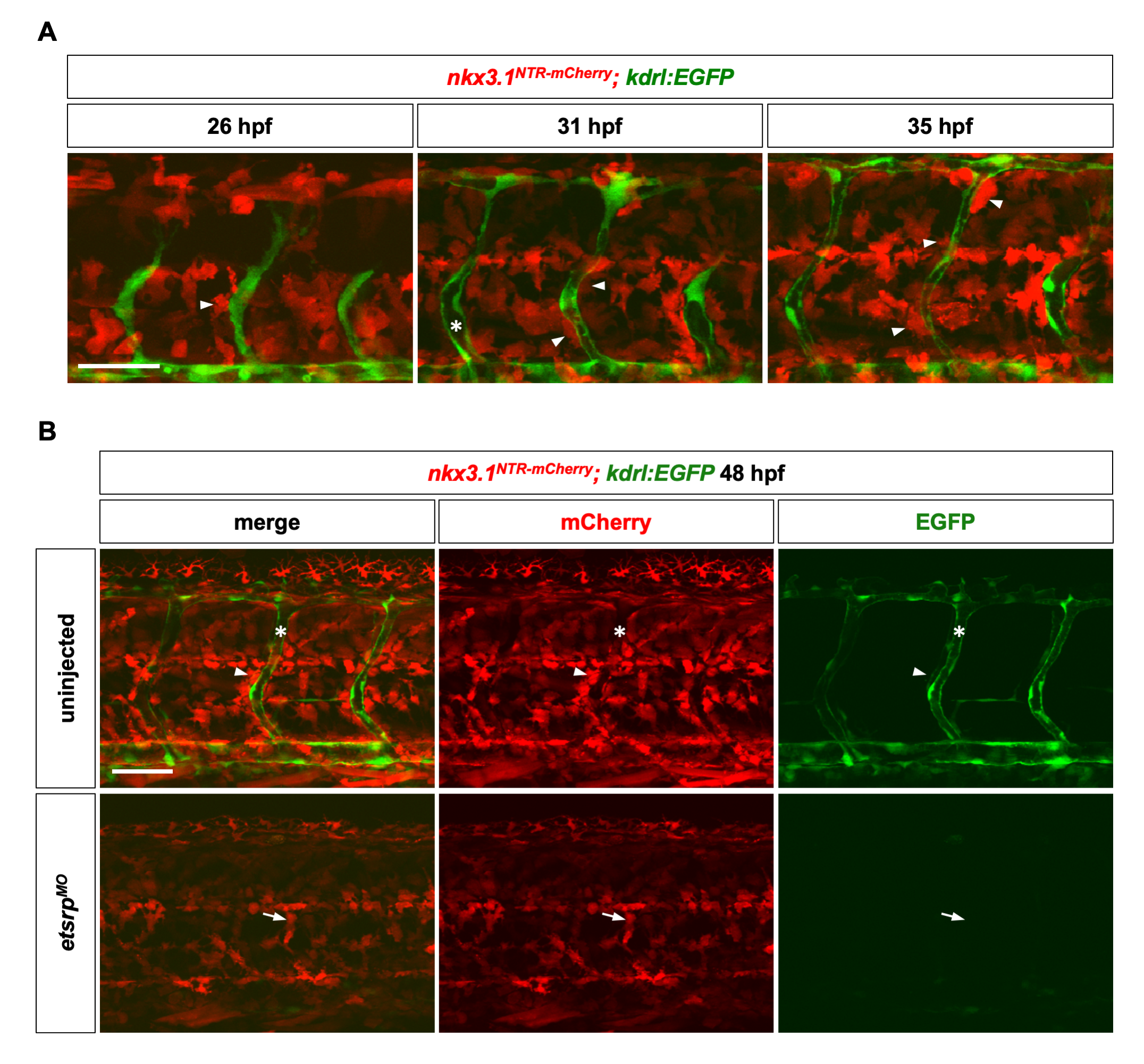

Supplement: S2 Fig — (A) Developmental time-course of perivascular fibroblasts. nkx3.1NTR-mCherry; kdrl:EGFP embryos were imaged at 26, 31, and 35 hpf to visualize different stages of perivascular fibroblast development. At 26 hpf, some nkx3.1NTR-mCherry cells (arrowhead) were visible along the ventral half of ISV sprouts. As ISV lumenization became visible (asterisk) at 31 hpf, more nkx3.1NTR-mCherry cells (arrowheads) appeared along ISVs. By 35 hpf, mCherry+ cells (arrowheads) were present along the entire length of ISVs. (B) nkx3.1NTR-mCherry; kdrl:EGFP embryos were injected with etsrp morpholino (etsrpMO) at the one-cell stage to block blood vessel formation. Representative images of uninjected control embryos (top) and etsrpMO injected morphants (bottom) at 48 hpf showing the distribution of sclerotome derived cells (red) in the presence and absence of ISVs (green), respectively. Trunk ISVs (asterisks) were visible in control embryos (top) but absent in morphants (bottom). Uninjected embryos had numerous mCherry+ perivascular fibroblasts (arrowheads), while morphants showed many mCherry+ sclerotome derived interstitial cells of unclear identity in the trunk (arrows). n = 15 (uninjected) and 23 (etsrpMO) embryos. Scale bars: 50 μm. (TIF) [file pgen.1008800.s002.tif]

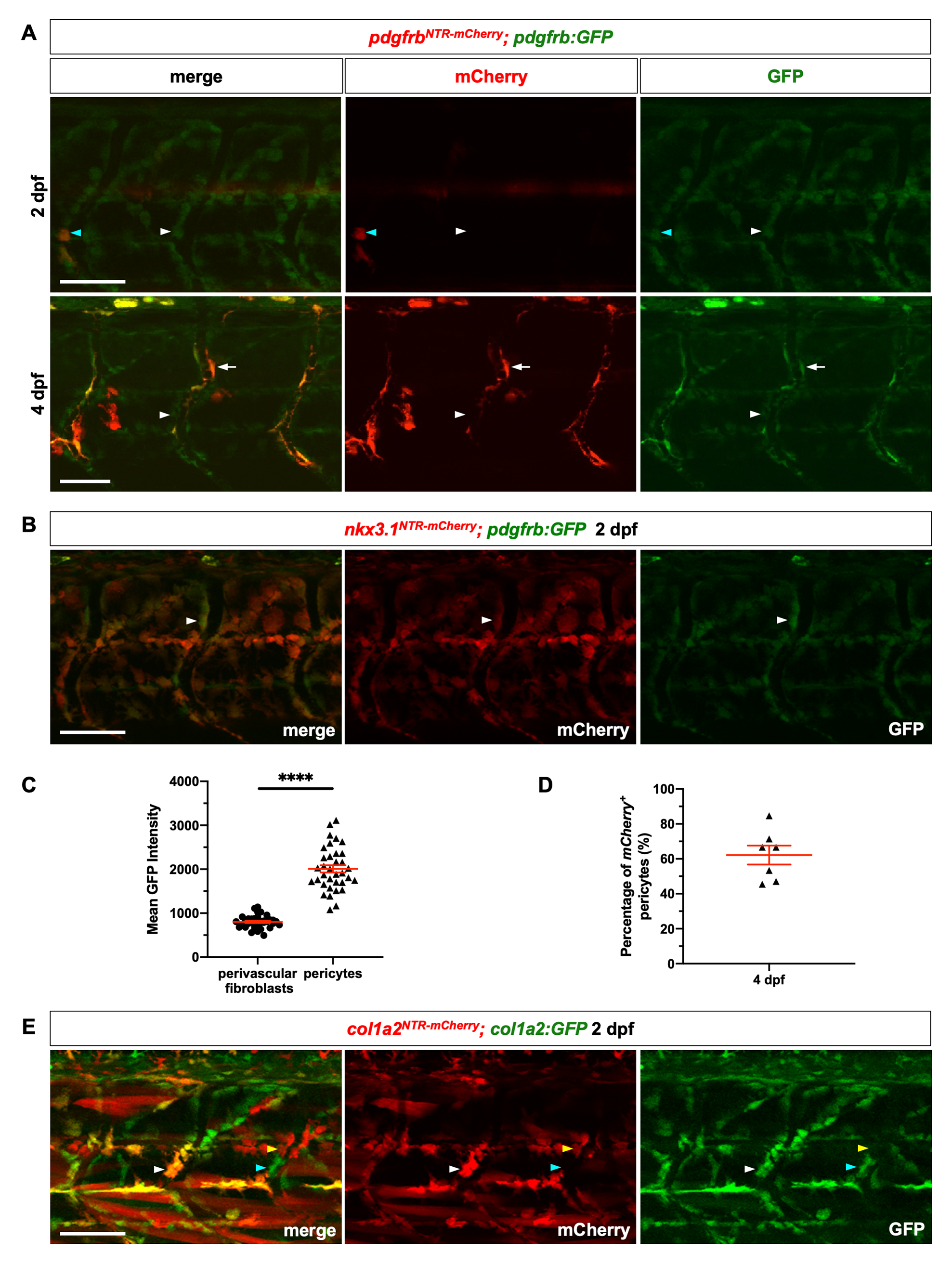

Supplement: S3 Fig — (A) pdgfrbNTR-mCherry; pdgfrb:GFP embryos imaged at 2 dpf (top) and 4 dpf (bottom). At 2 dpf, most perivascular fibroblasts were GFP+mCherry- (white arrowheads) while a few cells were GFP+mCherry+ (cyan arrowheads). At 4 dpf, pericytes were GFPhighmCherry+ (arrows), whereas perivascular fibroblasts were GFPlowmCherry- (arrowheads). n = 8 (2 dpf) and 7 (4 dpf) embryos. (B) nkx3.1NTR-mCherry; pdgfrb:GFP embryos imaged at 2 dpf. Perivascular fibroblasts (arrowheads) were positive for both nkx3.1NTR-mCherry (red) and pdgfrb:GFP (green) reporters. n = 15 embryos. (C) Quantification of pdgfrb:GFP expression in pericytes and perivascular fibroblasts in pdgfrbNTR-mCherry; pdgfrb:GFP embryos at 4 dpf from (A). GFP intensity was measured within individual GFPhighmCherry+ pericytes and GFPlowmCherry- perivascular fibroblasts using ImageJ. Pericytes showed 2.5 fold increase in GFP intensity compared to perivascular fibroblasts at 4 dpf. Data are plotted as mean ± SEM. n = 35 pericytes and 35 perivascular fibroblasts from 7 embryos. Statistics: Mann-Whitney U test. Asterisk representation: p-value < 0.0001 (****). (D) Quantification of the mosaicism of pdgfrbNTR-mCherry compared to the pdgfrb:GFP line. Total mCherry+ and GFPhigh pericytes were counted in pdgfrbNTR-mCherry; pdgfrb:GFP embryos at 4 dpf from (A), and double positive pericytes (GFPhighmCherry+) were graphed as a proportion of all GFPhigh pericytes. On average, the pdgfrbNTR-mCherry transgene labeled 62% of GFPhigh pericytes. Data are plotted as mean ± SEM. n = 7 embryos. (E) col1a2NTR-mCherry; col1a2:GFP embryos imaged at 2 dpf. Due to the mosaic nature of both reporters, some perivascular fibroblasts were GFP+mCherry+ (white arrowheads), some GFP+mCherry- (cyan arrowheads), and some GFP-mCherry+ (yellow arrowheads). n = 5 embryos. Scale bars: 50 μm. (TIF) [file pgen.1008800.s003.tif]

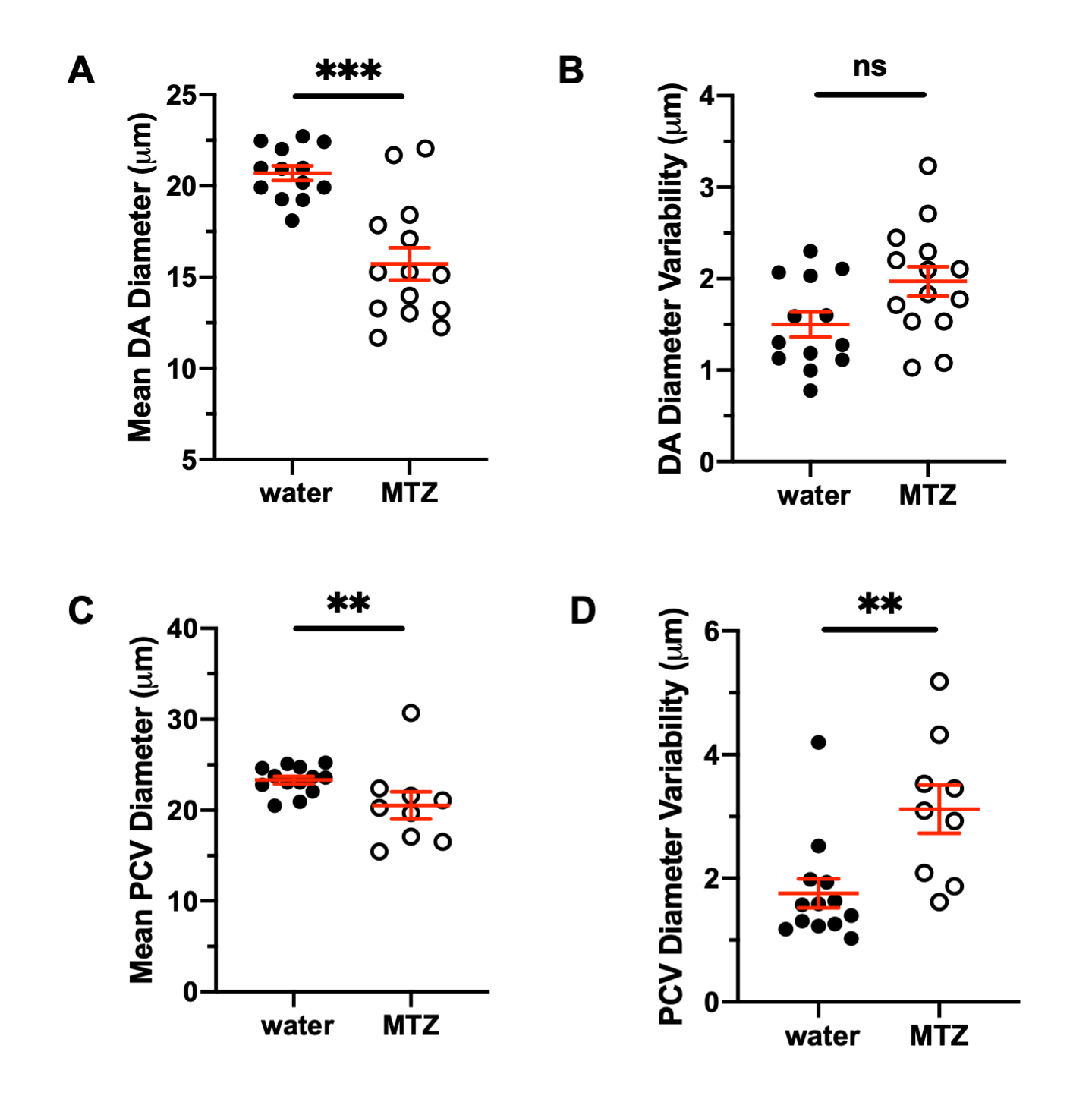

Supplement: S4 Fig — To examine the impact of perivascular fibroblast ablation on large trunk vessels, nkx3.1NTR-mCherry; kdrl:EGFP embryos were treated with either water or metronidazole (MTZ) from 38 to 62 hpf and then imaged as described in Fig 5A. Vessel diameters were measured at 6–10 points along each vessel using the line tool in ImageJ for both the dorsal aorta (DA) and the posterior cardinal vein (PCV). Mean diameter of each vessel and standard deviation from the mean (diameter variability) were plotted in (A-D). MTZ treated embryos showed reduced DA (A) and PCV (C) diameter and increased PCV diameter variability (D). DA diameter variability was not significantly different between MTZ treated and control embryos. n = 13 embryos (water); 9–14 embryos (MTZ). Results are graphed as mean ± SEM. Statistics: Mann-Whitney U test. Asterisk representation: p-value > 0.05 (ns, not significant); p-value < 0.01 (**); p-value < 0.001 (***). (TIF) [file pgen.1008800.s004.tif]

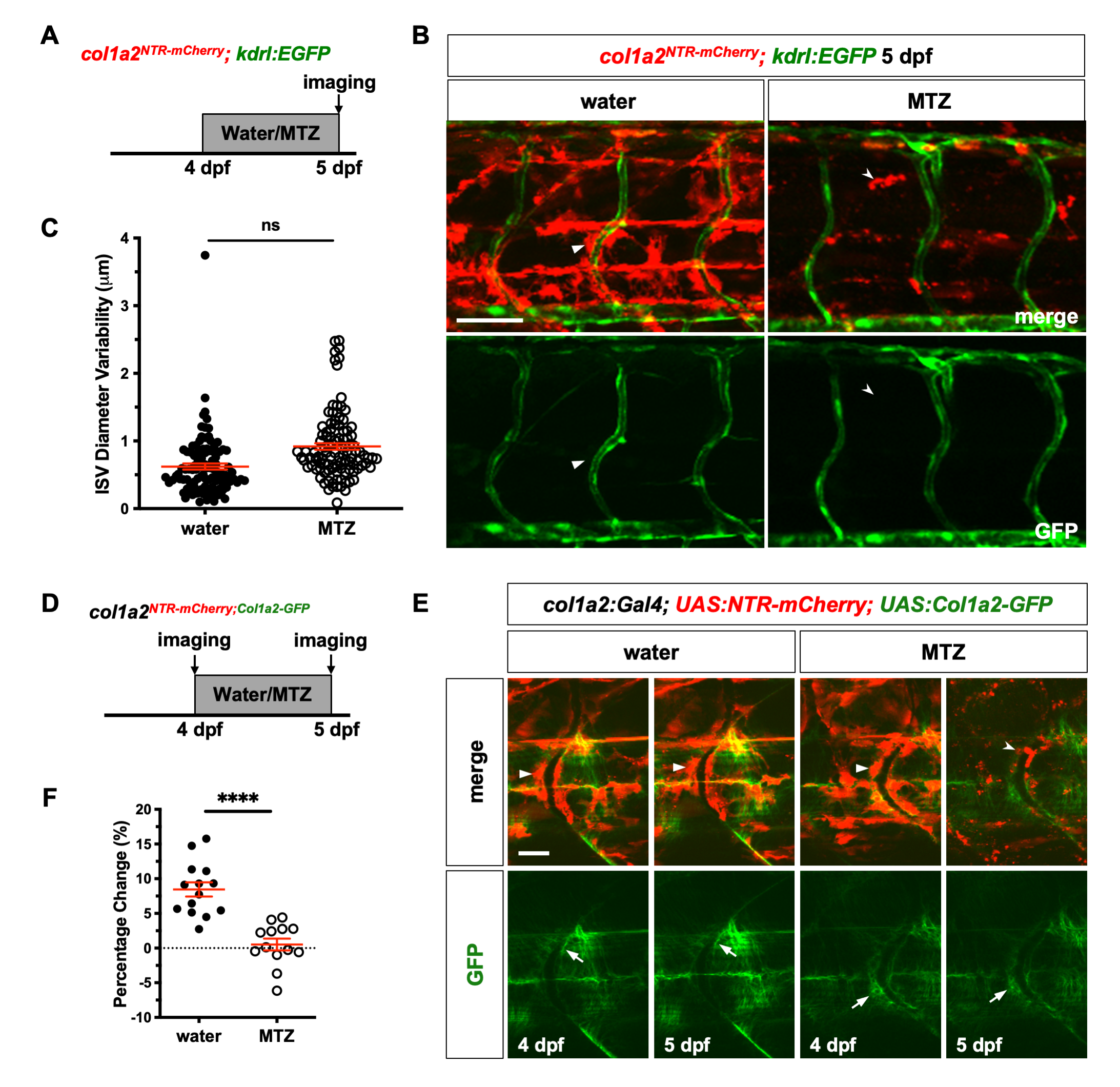

Supplement: S5 Fig — (A) Schematic of experimental procedure for late perivascular fibroblast ablation. col1a2NTR-mCherry; kdrl:EGFP embryos were incubated in either water or MTZ from 4 to 5 dpf and imaged to visualize ISV morphology. (B) Representative images showing water (left) and MTZ (right) treated embryos. Water-treated control embryos had many mCherry+ cells (arrowheads), whereas MTZ treatment resulted in complete perivascular fibroblast ablation, with only mCherry+ debris visible (notched arrowheads). No distinguishable difference in ISV morphology was visible between MTZ treated and control embryos. (C) Quantification of ISV diameter variability in (B). ISV diameter and variability measurements were quantified as described in Fig 5. n = 103 ISVs from 9 embryos (water); 107 ISVs from 13 embryos (MTZ). (D) Schematic of experimental protocol to examine collagen deposition after perivascular fibroblast ablation between 4 and 5 dpf. col1a2:Gal4; UAS:NTR-mCherry; UAS:Col1a2-GFP embryos were incubated in water or metronidazole (MTZ) from 4–5 dpf. The same mid-trunk region of individual embryos was imaged prior to and after the drug treatment to visualize Col1a2-GFP deposition. (E) Representative images of water (left) and MTZ (right) treated embryos before and after the drug treatment. Water-treated control embryos showed many mCherry+ cells (arrowheads), while MTZ treatment resulted in complete ablation of mCherry+ cells with only mCherry+ debris (notched arrowhead) remaining. Control embryos showed a slight increase in Col1a2-GFP deposition around ISVs (arrows) from 4 to 5 dpf, while MTZ treated embryos showed largely similar levels of Col1a2-GFP during the same time period. (F) Quantification of changes in fluorescence intensity of Col1a2-GFP in (E). GFP intensity was measured for each embryo before and after the drug treatment and percentage change in GFP intensity was calculated using the following formula: (GFPafter—GFPbefore) / GFPbefore x 100%. Control but not MTZ treated emb [file pgen.1008800.s005.tif]

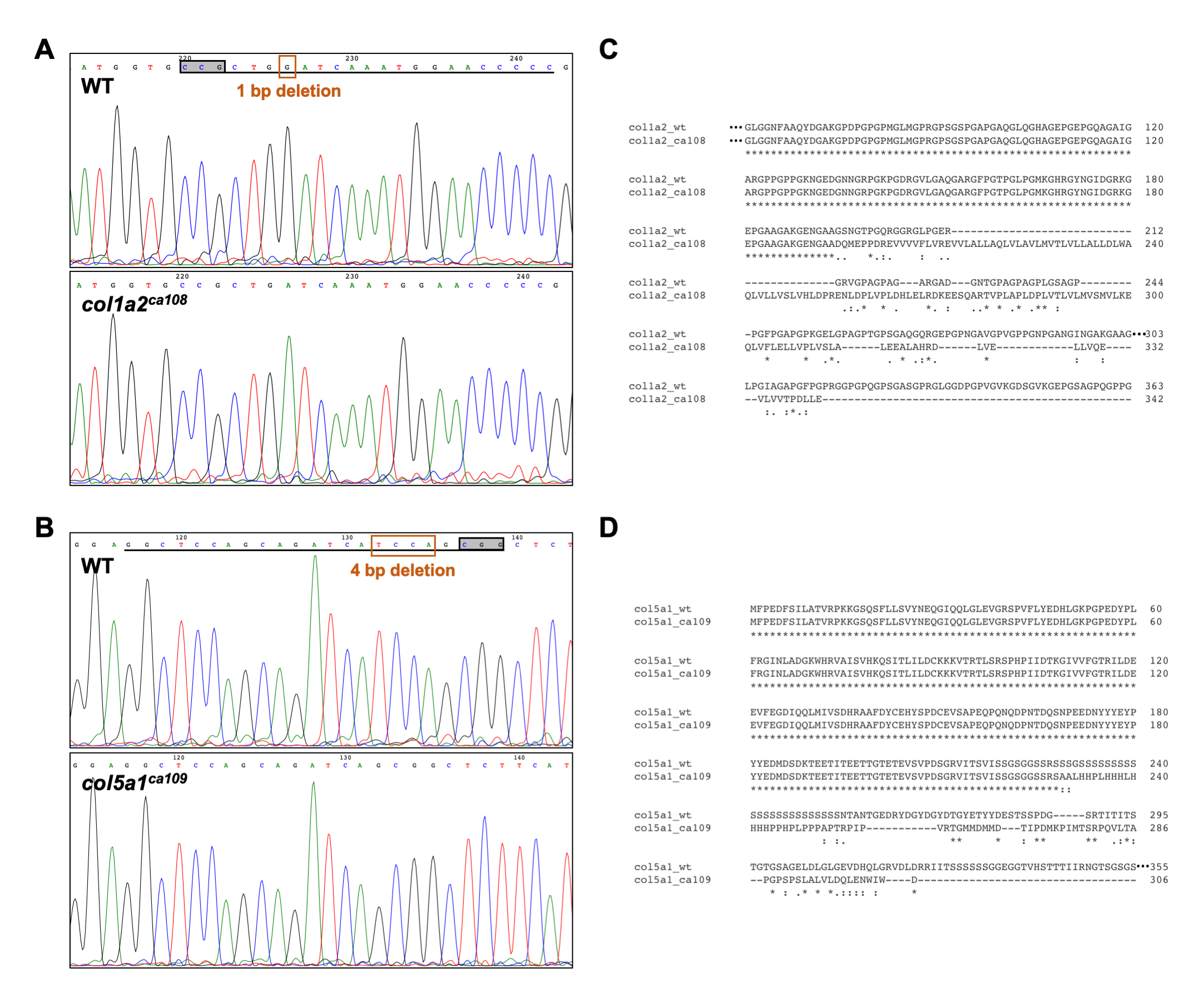

Supplement: S6 Fig — (A) Sequencing chromatograms of col1a2 WT and col1a2ca108 sequences. The sgRNA target sequence is underlined and the PAM motif is highlighted with a black box. The 1bp deletion in the col1a2ca108 sequence is denoted with an orange box. (B) Sequencing chromatograms of col5a1 WT and col5a1ca109 sequences. The sgRNA target sequence is underlined and the PAM motif is highlighted with a black box. The 4bp deletion in the col5a1ca109 sequence is denoted with an orange box. (C) Alignment of col1a2 WT and col1a2ca108 protein sequences. (D) Alignment of col5a1 WT and col5a1ca109 protein sequences. Protein sequences were aligned using Clustal Omega (https://www.ebi.ac.uk/Tools/msa/clustalo/). (TIF) [file pgen.1008800.s006.tif]

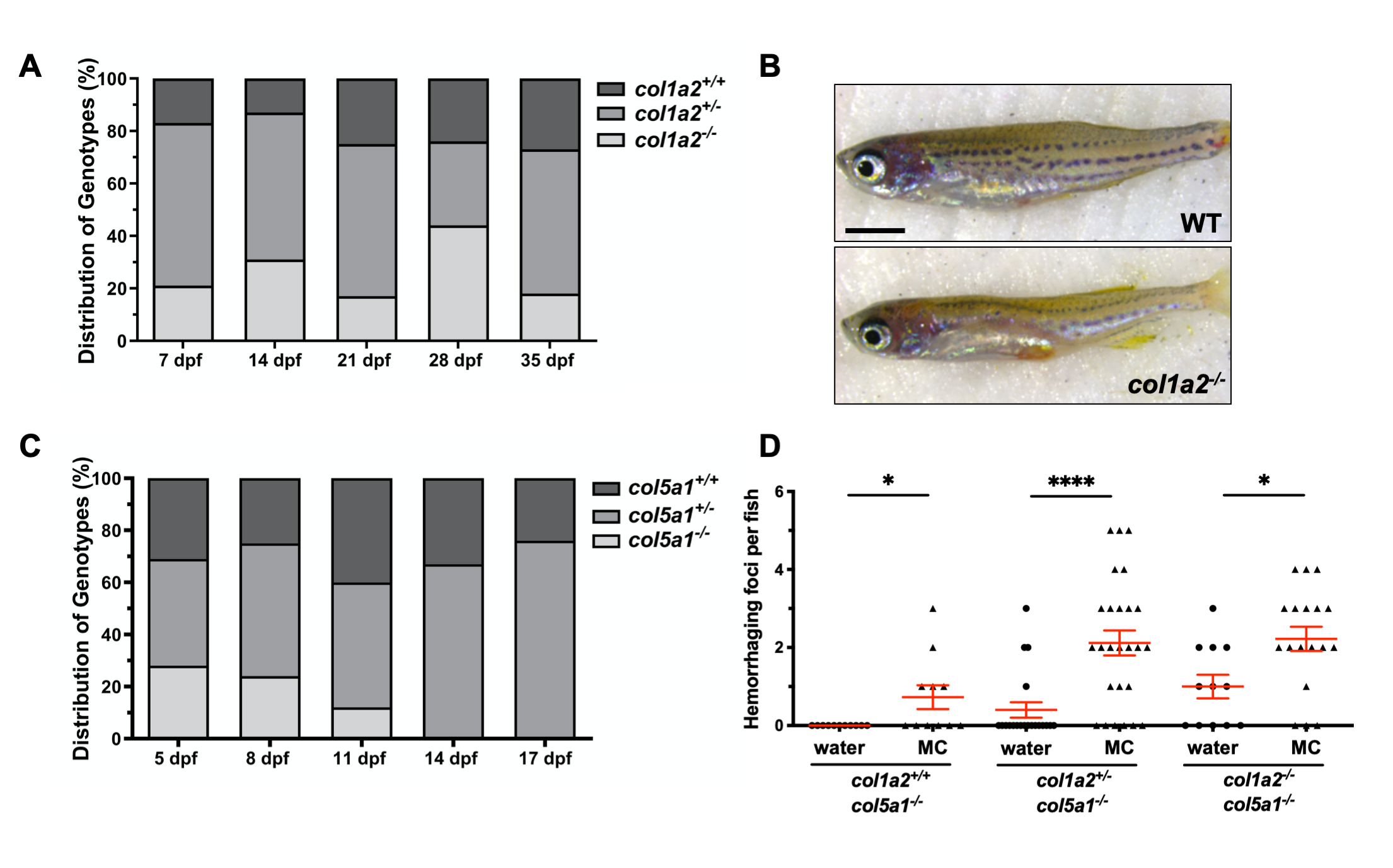

Supplement: S7 Fig — (A) Distribution of genotypes in progeny of col1a2+/- intercrosses. Embryos from the mentioned crosses were grown and genotyped every 7 days from 7 dpf to 35 dpf to determine genotype distribution. Distribution of genotypes followed roughly Mendelian ratios at all stages examined. n = 24 (7 dpf), 16 (14 dpf), 24 (21 dpf), 25 (28 dpf), and 22 (35 dpf) fish. (B) Comparison of adult wild type and col1a2-/- siblings. (C) Distribution of genotypes in progeny of col5a1+/- intercrosses. Embryos from crosses of col5a1+/- adults were grown and genotyped at 3 day intervals from 5 dpf to 17 dpf. While the distribution of genotypes followed Mendelian ratios at 5 and 8 dpf, col5a1-/- fish were completely absent at 14 and 17 dpf. n = 39 (5 dpf), 55 (8 dpf), 43 (11 dpf), 36 (14 dpf), and 32 (17 dpf) fish. (D) Quantification of hemorrhage severity in collagen mutants shown in Fig 6D and 6E. Hemorrhage severity was scored by counting the number of visible hemorrhage foci present in the trunk. Fish with no visible hemorrhage were counted as 0. Increased physical stress in the viscous MC solution resulted in an increase in hemorrhage severity across mutants with different genotypes. n = 12 (col1a2+/+; col5a1-/- + water); 11 (col1a2+/+; col5a1-/- + MC); 20 (col1a2+/-; col5a1-/- + water); 26 (col1a2+/-; col5a1-/- + MC); 12 (col1a2-/-; col5a1-/- + water); and 18 (col1a2-/-; col5a1-/- + MC) embryos. Results were graphed as mean ± SEM. Statistics: Mann-Whitney U test. Asterisk representation: p-value < 0.05 (*); p-value < 0.0001 (****). Scale bar: (B) 2 mm. (TIF) [file pgen.1008800.s007.tif]
